# The change of plasma metabolic profile and gut microbiome dysbiosis in patients with rheumatoid arthritis

**DOI:** 10.3389/fmicb.2022.931431

**Published:** 2022-10-18

**Authors:** Jing Zhu, Tingting Wang, Yifei Lin, Minghao Xiong, Jianghua Chen, Congcong Jian, Jie Zhang, Huanhuan Xie, Fanwei Zeng, Qian Huang, Jiang Su, Yi Zhao, Shilin Li, Fanxin Zeng

**Affiliations:** ^1^Department of Rheumatology and Immunology, West China Hospital, Sichuan University, Chengdu, China; ^2^Department of Rheumatology and Immunology, Sichuan Provincial People’s Hospital, University of Electronic Science and Technology of China, Chengdu, China; ^3^Department of Clinical Research Center, Dazhou Central Hospital, Dazhou, China; ^4^Precision Medicine Center, West China Hospital, Sichuan University, Chengdu, China; ^5^North Sichuan Medical College, Nanchong, China; ^6^School of Basic Medical Sciences, Chengdu University of Traditional Chinese Medicine, Chengdu, China; ^7^Sichuan Province Orthopaedic Hospital, Chengdu, China; ^8^Dazhou Vocational and Technical College, Dazhou, China; ^9^Frontiers Science Center for Disease-Related Molecular Network, West China Hospital, Clinical Institute of Inflammation and Immunology, Sichuan University, Chengdu, China

**Keywords:** rheumatoid arthritis, metabolomics, gut bacterial, gut fungus, metabolic pathway

## Abstract

**Objective:**

Rheumatoid arthritis (RA) is a chronic inflammatory joint disease, which is associated with progressive disability, systemic complications, and early death. But its etiology and pathogenesis are not fully understood. We aimed to investigate the alterations in plasma metabolite profiles, gut bacteria, and fungi and their role of them in the pathogenesis of RA.

**Methods:**

Metabolomics profiling of plasma from 363 participants including RA (*n* = 244), systemic lupus erythematosus (SLE, *n* = 50), and healthy control (HC, *n* = 69) were performed using the ultra-high performance liquid chromatography-quadrupole time-of-flight mass spectrometry. The differentially expressed metabolites were selected among groups and used to explore important metabolic pathways. Gut microbial diversity analysis was performed by 16S rRNA sequencing and ITS sequencing (RA = 195, HC = 269), and the specific microbial floras were identified afterward. The diagnosis models were established based on significant differential metabolites and microbial floras, respectively.

**Results:**

There were 63 differential metabolites discovered between RA and HC groups, mainly significantly enriched in the arginine and proline metabolism, glycine, serine, and threonine metabolism, and glycerophospholipid metabolism between RA and HC groups. The core differential metabolites included L-arginine, creatine, D-proline, ornithine, choline, betaine, L-threonine, LysoPC (18:0), phosphorylcholine, and glycerophosphocholine. The L-arginine and phosphorylcholine were increased in the RA group. The AUC of the predictive model was 0.992, based on the combination of the 10 differential metabolites. Compared with the SLE group, 23 metabolites increased and 61 metabolites decreased in the RA group. However, no significant metabolic pathways were enriched between RA and SLE groups. On the genus level, a total of 117 differential bacteria genera and 531 differential fungal genera were identified between RA and HC groups. The results indicated that three bacteria genera (*Eubacterium_hallii_group, Escherichia-Shigella, Streptococcus*) and two fungal genera (*Candida* and *Debaryomyces*) significantly increased in RA patients. The AUC was 0.80 based on a combination of six differential bacterial genera and the AUC was 0.812 based on a combination of seven differential fungal genera. Functional predictive analysis displayed that differential bacterial and differential fungus both were associated with KEGG pathways involving superpathway of L-serine and glycine biosynthesis I, arginine, ornithine, and proline interconversion.

**Conclusion:**

The plasma metabolism profile and gut microbe profile changed markedly in RA. The glycine, serine, and threonine metabolism and arginine and proline metabolism played an important role in RA.

## Introduction

Rheumatoid arthritis (RA) is a systemic autoimmune disease characterized by inflammation, especially persistent synovitis, and progressive joint damage with dominant extra-articular features (Smolen et al., 2016). It affects up to 1% of the general population worldwide, regardless of age group (Smolen et al., 2018). Although strict control and targeted therapy may retard the progress of RA, it still cannot be cured completely (Smolen et al., 2020).

With the rapid development of high-throughput technologies, including mass spectrometry (MS) and nuclear magnetic resonance spectroscopy (NMRS) (Kang et al., 2015), metabolomics was initiated recently to have exceptional advantages on some novel metabolic pathways and related metabolites (Zhang et al., 2018). Since metabolites are a specific manifestation of the metabolic process, they are indicative of a certain disease state (Horgan and Kenny, 2011). A number of previous studies have claimed the potential value of metabolites for RA (Chimenti et al., 2013; Kapoor et al., 2013; Priori et al., 2015; Cuppen et al., 2016). For example, arachidonic acid metabolism, sphingolipid metabolism, and arginine and proline metabolism were found essential in the therapeutic response of RA, due to achieving sustained drug-free remission (Hu et al., 2011; Teitsma et al., 2018; Wang et al., 2021). RA and systemic lupus erythematosus (SLE) are common autoimmune diseases. In fact, these two autoimmune diseases share several clinical manifestations, serological profiles, and immunological characteristics. However, differences in the plasma metabolic profile between RA and SLE groups are rarely reported.

Due to biological coevolution, flora metabolism has also been found to affect human health through “functional acquisition.” Numerous pathological changes are found accompanied (Grover and Kashyap, 2014; Foster et al., 2016; Tang et al., 2020). Since the significant role of intestinal flora in RA was first identified in 1968 (Olhagen and Mansson, 1968), it was further considered to be involved in potential treatment. For instance, the diversity of gut microbiota is found to decrease in RA patients (Smolen et al., 2018), while the rare taxa, that is, *Actinobacteria* increased (Chen et al., 2016). The *prevotella copri* was more common in new-RA patients than in established RA or no-RA patients (Scher et al., 2013). Thus, the gut microbiota is also suggested to be potential markers of the development of RA.

However, most studies only focused on the correlation of the RA with either a certain specific metabolite or microbial flora (Song et al., 2020; Mun et al., 2021). Very few of them can combination both plasma and fecal metabolomics to comprehensively explore their correlation with RA, not to mention constructing a predictive model to predict the development of RA. Therefore, we aimed to investigate changes in plasma metabolite profiles, gut bacteria, and fungi, and to explore their role in the pathogenesis of RA.

## Materials and methods

### Study patients

**FIGURE 1:**
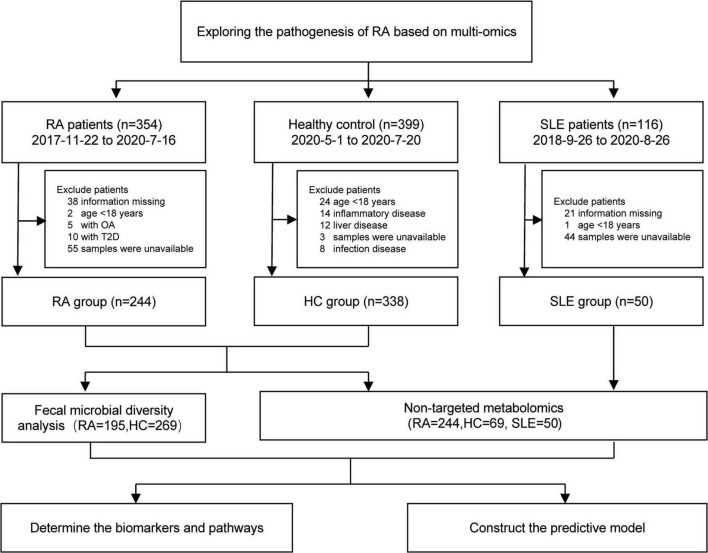
The flowchart of the study. RA, Rheumatoid arthritis; HC, healthy control; SLE, systemic lupus erythematosus.

The flow chart of this study is shown in Figure 1. The RA patients were diagnosed according to the American College of Rheumatology 2010 classification (Kay and Upchurch, 2012), hospitalized in the Department of Rheumatology and Immunology in Dazhou Central Hospital from November 2017 to July 2020. The RA group exclusion criteria are as follows: (1) Age < 18 years old; (2) Combination other immune metabolic diseases and complications, such as diabetes, osteoarthritis, metabolic syndrome, and infection; (3) Used probiotics and antibiotics in the last 2 weeks; (4) Tumor patients; (5) Organ failure and organ transplant patients; and (6) Pregnant women. The collected data included patients’ age, gender, 28-joint tender joint count (TJC28), 28-joint swelling joint count (SJC28), C-reactive protein (CRP), erythrocyte sedimentation rate (ESR), and so on.

The healthy control (HC) group included 338 participants, who underwent health examination in Dazhou Central Hospital from May 2020 to July 2020. The disease control group included 50 SLE patients hospitalized in Dazhou Central Hospital from September 2018 to August 2020. All SLE patients were diagnosed according to the American College of Rheumatology (ACR’97) and/or Systemic Lupus International Collaborating Clinic (SLICC’12) classification criteria (Hochberg, 1997; Petri et al., 2012). The plasma samples from 363 participants (244 RA, 69 HC, 50 SLE) were analyzed by non-targeted metabolomics.

The stool samples from 464 participants (195 RA and 269 HC) were used to identify the microbial biomarkers and construct the predictive model.

This study was approved by the Ethics Committee of Dazhou Central Hospital. The subjects have signed informed consent.

### Plasma sample collection

Four milliliters of fasting venous blood were drawn and placed in an anticoagulant vacuum blood collection tube (BD Vacutainer^®^). After the blood was collected, the samples were placed in a refrigerator at 4°C to stand still for processing within 2 h after collection. First, each tube of blood was centrifuged at 3,500 rpm, 4°C for 10 min, and the supernatant was separated into two EP tubes, and then centrifuged again at 12,000 rpm, 4°C for 10 min. Finally, the supernatant plasma was transferred into two new EP tubes and stored in the refrigerator at -80°C.

### Untargeted metabolomics analysis by UNPLC/Q-TOF-MS

The method detail of UNPLC/Q-TOF-MS was shown in Supplementary Method File. The hierarchical cluster was performed to display the relationship and difference between metabolites. The multidimensional statistical analysis, including principal component analysis (PCA), partial least-squares discriminant analysis (PLS-DA), and orthogonal partial least-squares discriminant analysis (OPLS-DA), were also performed to help cluster the metabolites. Besides, a permutation test was performed for the validation of the model. Metabolic pathways [impact < 0.2, −log10(*p*) value > 1.3] and greater metabolic abundance were identified by KEGG database.

### Fecal sample collection

The fresh stool was collected and delivered immediately at low temperatures, then divided into individual parts of 200 mg and stored at −80°C until extraction.

### Fecal sample DNA extraction and Illumina MiSeq sequencing

According to the manufacturer’s protocol, total DNA was extracted from fecal samples. Bacterial 16S rRNA gene fragments (V3–V4) were amplified from the extracted DNA using the primers 338F ACTCCTACGGGAGGCAGCAG and 806R GGACTACHVGGGTWTCTAAT, and fungal internally transcribed spacer (ITS) gene fragments were amplified from the extracted DNA using the primers ITS1F CTTGGTCATTTAGAGGAAGTAA and ITS2R GCTGCGTTCTTCATCGATGC.

The following PCR Amplicons were subjected to paired-end sequencing on an Illumina MiSeq sequencing platform using a PE250 kit.

### Amplification sequence processing and analysis

Taxonomic assignment of ASVs was performed using the Naive Bayes consensus taxonomy classifier implemented in Qiime2 and the SILVA 16S rRNA and ITS database. ASV analysis, Community diversity analysis, Genus and Species difference analysis, model predictive analysis, and PICRUSt2 (Phylogenetic Investigation of Communities by Reconstruction of Unobserved States 2) function prediction analysis were performed based on the 16S rRNA bacterial and ITS Fungal sequencing data.

The method of microbial diversity analysis is detailed in Supplementary Method File.

### Statistical analysis

The One-Way ANOVA and *t*-test were performed using SPSS Statistics (V.24.0.0.0) (SPSS Inc., Chicago, United States). The bar graph was performed using GraphPad Prism (v6.0) (GraphPad Software, Inc., CA, United States). And the Principal Co-ordinates Analysis, PCA, Phylogenetic Investigation of Communities by Reconstruction of Unobserved States, Receiver Operating Characteristic (ROC) curve, OPLS-DA, and Correlation heat map analysis were performed using R software (Version 3.4.4).

## Results

### Baseline characteristics of healthy control, systemic lupus erythematosus, and rheumatoid arthritis participants

The baseline characteristics of the three groups are presented in Table 1. The RA group included 244 patients (age, 57.8 ± 12.7 years; 72.5% women), with average DAS28(3) 5.5 ± 1.5. The HC group included 338 cases (age, 49.3 ± 7.7 years; 18.6% women). The disease control SLE group included 50 cases (age, 46.2 ± 9.4 years; 98.0% women). There are significant differences in the other clinical indicators of the three groups, such as white blood cells, neutrophils, and lymphocytes (all *p* < 0.0001).

**TABLE 1:** The characteristics of the patients with RA or SLE and healthy controls.

Parameters	HC (*n* = 338)	RA (*n* = 244)	SLE (*n* = 50)	*P*
Age (y)	49.3 ± 7.7	57.8 ± 12.7	46.2 ± 9.4	<0.0001
Sex, F, No. (%)	63 (18.6)	177 (72.5)	49 (98)	<0.0001
RF (U/ML)	–	300.9 ± 312.3	26.0 ± 91.0	<0.0001
DAS28(3)	–	5.5 ± 1.5	–	–
CRP (mg/L)	–	43.2 ± 40.0	4.8 ± 5.8	<0.0001
ESR (mm/h)	–	68.7 ± 30.2	25.5 ± 17.1	<0.0001
TJC28	–	9.9 ± 8.8	–	–
SJC28	–	8.0 ± 8.0	–	–
WBC (10^9^/L)	5.3 ± 1.3	7.2 ± 2.9	5.8 ± 2.6	<0.0001
NEUT (10^9^/L)	3.2 ± 1.0	5.2 ± 2.6	2.7 ± 2.0	<0.0001
LY (10^9^/L)	1.6 ± 0.4	1.3 ± 0.6	31.0 ± 32.9	<0.0001

The *p*-value < 0.05 means a significant difference between groups. RA, Rheumatoid arthritis; HC, healthy control; RF, rheumatoid factor; DAS28, Disease Activity Score 28; CRP, C-reactive protein; ESR, Erythrocyte Sedimentation Rate; TJC28, Tender 28-joint count; SJC28, Swollen 28-joint count; WBC, white blood cell; NEUT, Neutrophil; LY, lymphocyte.

### Plasma metabolomics profiles of healthy control, systemic lupus erythematosus, and rheumatoid arthritis participants

Based on an untargeted metabolomics analysis by UNPLC/Q-TOF-MS, we detected 486 peaks in positive and negative ion modes. After excluding 62 metabolites by the natural isotopic peaks, the rest 424 metabolites were finally included for analysis. The PCA results of metabolites indicated that HC group could be distinguished markedly from RA and SLE groups, whereas SLE and RA groups showed less obvious separation (Supplementary Figures 1A,B). Results of the permutation test were shown in Supplementary Figures 1C,D. A total of 63 differential metabolites were identified between RA and HC groups, 21 of which increased and 42 of which decrease in the RA group compared with the HC group (Supplementary Figures 1E,F).

**FIGURE 2:**
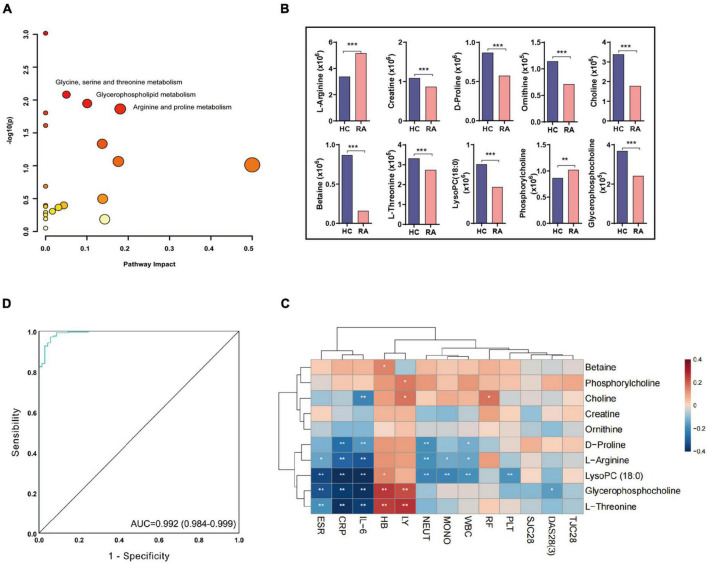
The biomarkers and pathway in RA based on plasma metabolomics. **(A)** The three significant pathways are based on 10 differential metabolites between RA and HCs (Pathway impact < 0.2, −log10(*p*) value > 1.5 and greater metabolic abundance). **(B)** The metabolite content differences between RA and HC. *P < 0.05, **P < 0.01, ***P ≤ 0.001. **(C)** The correlation heatmap between metabolites and clinical information. **(D)** The ROC curve of the combination of 10 differential metabolites for classifying RA patients from HC. RA, rheumatoid arthritis; HC, healthy control; DAS28, disease activity score-28; TJC28, tender joint count 28; SJC28, joint swelling count 28; ESR, erythrocyte sedimentation rate; CRP, C-reactive protein; RF, rheumatoid factor; WBC, white blood cell count; LY, lymphocyte count; NEUT, neutrophil count; MONO, monocyte count; HB, hemoglobin; PLT, platelet count, ROC, receiver operating characteristic.

After KEGG pathway analysis, there were three metabolic pathways that most significantly changed between RA and HC groups, arginine and proline metabolism, glycine, serine and threonine metabolism, and glycerophospholipid metabolism (Figure 2A). Among them, a total of 10 differential metabolites were discovered, including L-arginine, creatine, D-proline, ornithine, choline, betaine, L-threonine, 1-stearoyl-2-hydroxy-sn-glycero-3-phosphocholine [LysoPC(18:0)], phosphorylcholine, and glycerophosphocholine (Figure 2B and Supplementary Table 1). The L-arginine and phosphorylcholine were increased in the RA group (all *p* < 0.01). The correlation heatmap indicated that the patient’s disease activity [DAS28(3)] was negatively correlated with glycerophosphocholine, and inflammation indicators (IL-6, CRP) were negatively correlated with D-proline, L-arginine, L-threonine, LysoPC (18:0), and glycerophosphocholine (Figure 2C). The result of the ROC analysis indicated that the predictive model showed a high discriminatory power to predict the RA status and the area under the curve (AUC) was 0.992 based on a combination of the 10 differential metabolites (Figure 2D).

**FIGURE 3:**
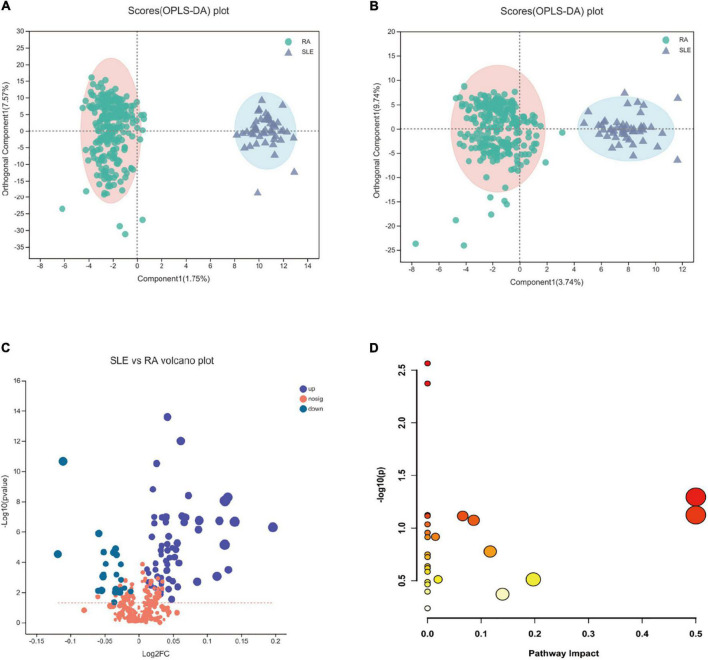
The orthogonal partial least-squares discriminant analysis (OPLS-DA) and KEGG pathway analysis in RA patients compared to SLE patients. **(A,B)** The OPLS-DA plots between RA and SLE, cation, and anion, respectively. **(C)** The volcano plot of RA vs. SLE. **(D)** The KEGG pathway analysis results based on the significant metabolites between RA and SLE. RA, rheumatoid arthritis; SLE, systemic lupus erythematosus.

Compared with the disease control, the RA and SLE groups were distinguished significantly in the OPLS-DA analysis (Figures 3A,B). A total of 84 differential metabolites were identified by the differentiated analysis (Figure 3C). Compared with the SLE group, the difference analysis result displayed 23 metabolites increased in RA patients, such as deoxycholic acid, d-galacturonic acid, and L-aspartate. The other 61 metabolites were lower in the RA group, including L-glutamine, L-threonine, and L-alanine. However, all the fold changes of differential metabolites were 0.9–1.2, and no significant metabolic pathway was enriched in the KEGG pathway analysis based on all differential metabolites between RA and SLE groups (Figure 3D).

### Gut microbiota changes of rheumatoid arthritis participants

Fecal samples were obtained from 464 human participants, including 195 RA cases and another 269 HC volunteers. Bacterial 16S rRNA and fungal ITS amplification provided a series of ASVs to analyze the difference between the two groups.

#### 16S rRNA bacterial community analysis

The clear boundary described the significant difference between RA and HC groups and proved that the number of sequencing samples was sufficient. On the species level, a total of 596 bacterial species were identified between the two groups, including 219 unique bacterial species in the RA group and 147 unique bacterial species in HC (Supplementary Figure 2A). The beta of principal coordinates analysis (PCoA) suggested that there were significant differences in the horizontal community distribution of bacterial species between the two groups (Supplementary Figure 2B). By Wilcoxon rank-sum test, 169 differential bacterial species were identified, and the top 10 bacterial communities based on mean proportions were shown in Supplementary Figure 2C (*p* < 0.05). However, most names of differential specie names could not be identified.

**FIGURE 4:**
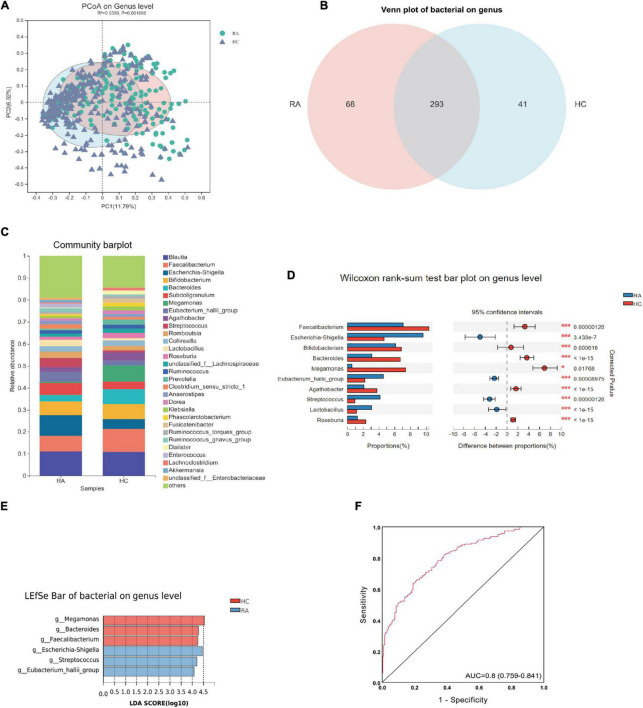
The differential bacterial flora and predicted enzymes based on 16s rRNA (genus level) between RA and HC groups. **(A)** The Venn plot between RA and HC groups. **(B)** The PCoA analysis between RA and HC. **(C)** The Bar graph of bacterial flora composition in RA and HC. **(D)** The top 10 significant bacteria between RA and HC. **(E)** The LDA bar of bacterial flora in RA and HC. **(F)** The ROC curve of the combination of six differential bacteria for classifying RA patients from HC. RA, rheumatoid arthritis; HC, healthy control; ROC, receiver operating characteristic.

On the genus level, there were 293 common bacterial genera between the two groups, 68 unique genera in the RA group and 41 unique genera in the HC group (Figure 4A). The PCoA analysis displayed that there were differences in the distribution of bacterial communities between the two groups (Figure 4B). The dominant bacteria genus in RA group includes *Blautia* (11.0%), *Faecalibacterium* (7.1%), *Escherichia-Shigella* (9.6%), *Bifidobacterium* (6.1%), *Subdoligranulum* (5.4%); the dominant bacteria genera in the HC group were *Blautia* (10.8%), *Faecalibacterium* (10.4%), *Bifidobacterium* (6.9%), *Bacteroides* (6.7%), and *Megamonas* (7.4%) (Figure 4C). A total of 117 differential bacteria genera were identified between groups by Wilcoxon rank-sum test, and the top 10 bacterial communities base on mean proportions were shown in Figure 4D (*p* < 0.05). Furthermore, the results of linear discriminant analysis effect size (LEfSe) identified that six key differential bacteria genera were screened again between groups (LDA score > 4.0 and *p* < 0.05), **including** *Eubacterium_hallii_group*, *Escherichia-Shigella*, *Megamonas*, *Bacteroides*, *Faecalibacterium*, and *Streptococcus* (Figure 4E). Among them, *Eubacterium_hallii_group, Escherichia-Shigella, and Streptococcus* increased in RA patients. Hence, the six most important differential bacterial genera were selected for predictive model analysis, and the AUC was 0.80 of the ROC curves (Figure 4F).

#### Fungal internally transcribed spacer analysis

On the fungal species level, there were 686 common fungal species between the two groups, 980 unique fungal species in the RA group, and 311 unique fungal species in HC group (Supplementary Figure 3A). The PCoA analysis on the species level showed that there were significant differences in the horizontal community distribution between the two groups (Supplementary Figure 3B). Using Wilcoxon rank-sum test, a total of 160 differential fungal species were identified; the top 15 fungus communities based on mean proportions are shown in Supplementary Figure 3C (*p* < 0.05).

**FIGURE 5:**
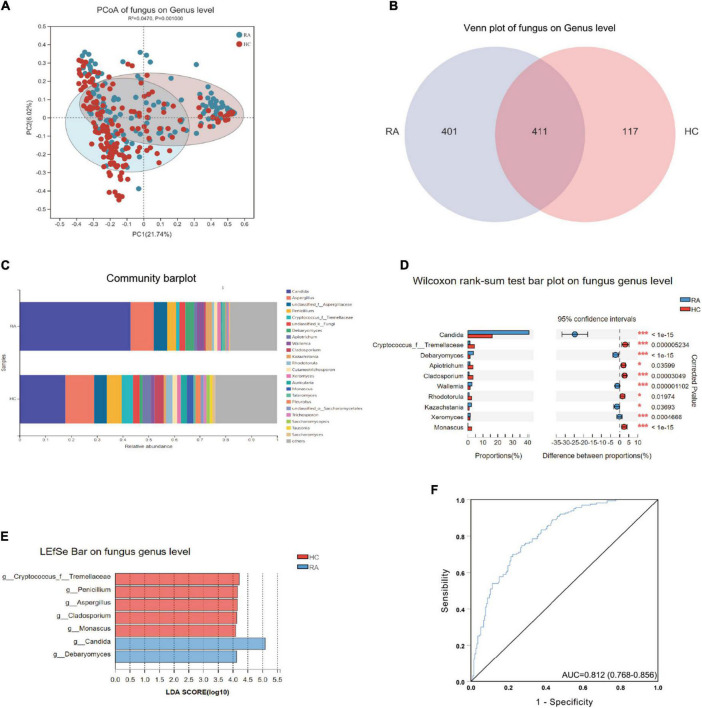
The differential fungal flora and predicted enzymes based on ITS (on genus level) between RA and HC. **(A)** The Venn plot between RA and HC groups. **(B)** The PCoA analysis between RA and HC groups. **(C)** The Bar graph of fungal flora composition in RA and HC groups. **(D)** The top 10 significant fungal between RA and HC groups. **(E)** The LDA bar of fungal flora in RA and HC groups. **(F)** The ROC curve of the combination of seven differential fungal for classifying RA patients from HC. RA, rheumatoid arthritis; HC, healthy control; ROC, receiver operating characteristic.

On the fungal genus level, the PCoA plot of beta diversity analysis showed that there were significant differences in the distribution of fungal communities between the two groups (Figure 5A). There were 411 common fungal genera between the two groups, 401 of which were specific fungal genera in the RA group, and 117 specific fungal genera in the HC group (Figure 5B). A total of 531 differential fungal genera were identified between RA and HC groups. Community bar diagram analysis showed that there were also significant differences in the fungal composition, relative abundance, and proportion between the two groups. The dominant fungal genera in the RA group were *Candida* (41.0%), *Aspergillus* (9.3%), *Debaryomyces* (3.7%), and *Penicillium* (3.4%); and the dominant fungus genera in the HC group were *Candida* (16.3%), *Aspergillus* (11.4%), *Penicillium* (5.9%), and *Cryptococcus_f_Tremellaceae* (4.4%) (Figure 5C). Using Wilcoxon rank-sum test, the top 10 fungal communities based on mean proportions are shown in Figure 5D. Specifically, *Cryptococcus, Apiotrichum, Cladosporium, Rhodotorula*, and *Monascus* were significantly more abundant in the HC group; and *Candida, Debaryomyces, Wallemia, Kazachstania*, and *Xeromycesis* were significantly more enriched in the RA group (*p* < 0.05). The LEfSe analysis result discovered that there were seven important different fungal genera between groups (LDA score > 4.0) including *Cryptococcus, Penicillium, Aspergillus, Cladosporium, Monascus, Candida*, and *Debaryomyces* (Figure 5E). Specifically, the *Candida* and *Debaryomyces* both increased in RA patients. The result of the ROC analysis suggested that the AUC of the prediction model was 0.812 based on a combination of the above seven differential fungal genera (Figure 5F).

### Functional predictive analysis

**FIGURE 6:**
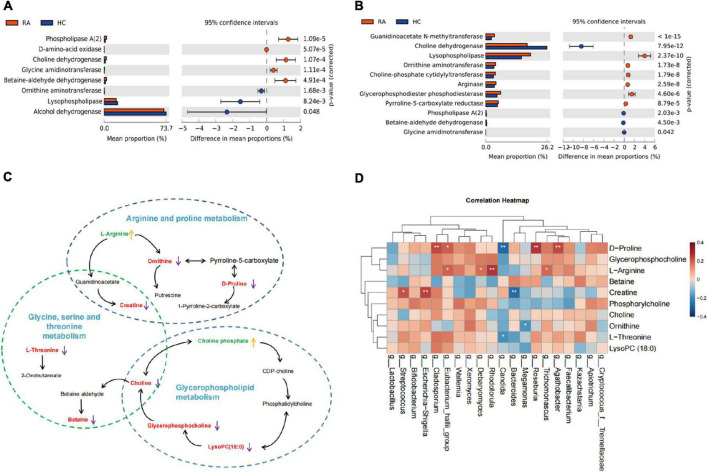
The function predictive and omics association analysis. **(A)** The enzymes prediction of bacterial metabolome between RA and HC. **(B)** The enzymes prediction of fugal metabolome between RA and HC. **(C)** The metabolic network diagram of the three enrichment pathways based on 10 differential metabolites. **(D)** The network mapping diagram of the differential metabolites, bacterial, and fungus. RA, rheumatoid arthritis; HC, healthy control.

We predicted changes in modules and pathways using PICRUSt2 and calculated the mean proportion of important enzymes in modules and pathways between the RA and HC groups using the STAMP software. PICRUSt2 analysis of the bacteria suggested that these changes in the relative ASV abundance might be associated with the regulation of pathways involving superpathway of L-serine and glycine biosynthesis I, arginine, ornithine, and proline interconversion (Supplementary Figure 4). The most important enzymes associated with the above pathways were predicted, namely choline dehydrogenase, D-amino-acid oxidase, glycine amidinotransferase, betaine-aldehyde dehydrogenase, ornithine aminotransferase, and lysophospholipase (Figure 6A). The functional predictive analysis of fungus also displayed that host metabolic pathways were regulated by fungi including superpathway of L-threonine biosynthesis, urea cycle, L-proline biosynthesis II (from arginine), superpathway of L-serine and glycine biosynthesis I (Supplementary Figure 5), and predictive enzymes including choline dehydrogenase, lysophospholipase, ornithine aminotransferase, choline-phosphate cytidylyltransferase, arginase, phospholipase A (2), and glycine amidinotransferase (Figure 6B). The metabolic network diagram revealed that the three KEGG pathways were enriched based on the 10 key differential metabolites between RA and HC groups (Figure 6C). The three metabolic pathways were interconnected by creatine and choline. Finally, the correlations between metabolites, bacteria, and fungi were analyzed. The results showed that changes in amino acid metabolism were associated with changes in gut microbes in RA patients. D-Proline concentration in plasma was significantly positively correlated with the abundance of *Agathobacter*, *Roseburia*, and *Cladosporium*, and negatively correlated with *Candida*. Among bacteria, we found that *Escherichia-Shigella* abundance was positively correlated with plasma Creatine concentration, while *Bacteroides* genus was negatively correlated with plasma Creatine concentration. The plasma L-Arginine concentration is increased in RA patients and is positively correlated with *Rhodotorula* of fungi in the stool (Figure 6D).

## Discussion

As RA is a chronic autoimmune disease, its etiology has not been fully understood yet. Genetic and environmental factors are important risk factors for disease incidence and development. In this study, we mainly focused on the metabolic disorder during RA disease progression using multi-omics approaches. We sought to apply plasma metabolomics profiling and gut microbial community profile to identify potential biomarkers for predicting RA-patient. The metabolomics analysis highlighted three main metabolic pathways: the glycine, serine, and threonine metabolism, glycerophospholipid metabolism, and arginine and proline metabolism. The AUC of the multivariate prediction models based on a combination of the 10 differential metabolites was 0.992. Additionally, the 16S rRNA sequencing results showed that the intestinal bacterial diversity and bacterial structure changed significantly in RA patients compared with the HC group. The AUC of the ROC curve was 0.80 to predict the RA, based on six core differential bacterial genera (LDA > 4). Further, the fungal diversity in RA patient group increased significantly more than in HC, and the AUC of the prediction model was 0.812 based on a combination of seven core differential fungal genera. Functional predictive analysis suggested that the superpathway of L-serine and glycine biosynthesis I, and arginine, ornithine, and proline interconversion might be regulated by bacterial and fungus. These results revealed that arginine and proline metabolism and glycine, serine, and threonine metabolism could play an important role in the disease incidence and development of RA.

The plasma metabolic changes, caused by the chronic inflammation, usually served as biomarkers for diagnosis (Chimenti et al., 2015). Our study showed that plasma levels of many kinds of amino acids (AAs) were reduced in RA patients, including L-tryptophan, L-alanine, L-threonine, and L-leucine, compared to healthy controls. These findings were similar to another study (Wang et al., 2018), which suggested that tryptophan (Trp) and glycine were decreased in patients with RA. These metabolites have been associated with immune system activation. For instance, serum alanine level was associated with synovial B-lymphocyte stimulator expression, while the serum levels of threonine and leucine were associated with synovial expression of IL-1β and IL-8 (Narasimhan et al., 2018). Trp, as one of the rarest essential AAs, might degrade to affect immunity through kynurenines and regulate T cells (Murray, 2016).

A large body of evidence indicated that arginine metabolism played an important role in the occurrence and development of RA disease (Radhakutty et al., 2017; Brunner et al., 2020). In our study, the plasma level of arginine was higher in RA patients than in HC by non-target metabolomics, which was also validated in target metabolomics analysis. This result is consistent with the previous study that displayed patients with RA had higher plasma concentrations of arginine (Radhakutty et al., 2017; Brunner et al., 2020). Brunner and his colleagues reported RANKL that cellular programming required extracellular arginine (Brunner et al., 2020). This evidence indicated that it could improve outcomes in murine arthritis models by systemic arginine restriction, and preosteoclast metabolic quiescence would be induced by arginine removal. It may be a possibility for the effective intervention of RA by arginine restriction. These studies suggested that the core AAs exhibited potential application value in the diagnosis, disease progression, and therapy of RA.

Choline, as a bridge of glycerophospholipid metabolism and glycine, serine, and threonine metabolism, was decreased in the blood of the RA group in our study. This is consistent with Rekha Narasimhan et al.’s study (Narasimhan et al., 2018). Choline C-11 PET scanning was transferred to joints in inflammatory arthritis (Roivainen et al., 2003) and increased in fibrocyte-like synoviocytes (Beckmann et al., 2015). Evidence of a previous research revealed that Choline was presented in synovial fibroblasts and associated with TNF-α production and migration (Guma et al., 2015). In our study, choline, L-threonine, and D-proline levels were inversely correlated with the RA disease activity defined by DAS28(3). This indicated that these plasma metabolites may be strongly linked to RA disease progression and serve as potential predictive markers.

RA and SLE are both typical chronic inflammatory autoimmune diseases, and with complicated pathogenesis. It is difficult to identify and clarify accurate etiology for them. Many previous studies have reported that abnormal metabolic activities are critical in SLE pathogenesis. For example, glycolysis and mitochondrial oxidative metabolism both were raised in SLE patients and the SLE mouse model (Yin et al., 2015). Another paper revealed that citrate and pyruvate were decreased in both SLE and RA patients compared with healthy controls, and the serum level of formate was markedly decreased in SLE patients (Ouyang et al., 2011). In our study, we compared the RA and SLE and found that the RA metabolic profile was different from the SLE group. However, there was no significant change in metabolic pathways between RA and SLE groups. Additionally, the fold change value of all differential metabolites was small (0.9-1.2), which may support a hypothesis that the changes in the plasma metabolic are similar for RA and SLE patients.

In recent years, numerous studies proved that there were alterations in intestinal microbiota composition, especially in autoimmune diseases (Wu and Wu, 2012; Luckey et al., 2013; Taneja, 2014), including RA. Previous studies showed that gut microbiome dysbiosis could induce the production of proinflammatory cytokines, interleukin-17, and increased levels of Th17 cells (Luckey et al., 2013). The role of gut microbiota in the pathogenesis of arthritis was demonstrated in experimental murine models (Chen et al., 2016; Liu et al., 2016; Wang et al., 2020a). For instance, *L. bifidus* could induce joint swelling in germ-free mice (Abdollahi-Roodsaz et al., 2008). In our study, we identified 68 genera unique in the RA group and 41 unique genera in the HC group, and 117 differential bacterial genera were further identified. On the genus level, the *Lactobacillus*, *Eubacterium_hallii, Escherichia-Shigella*, and *Streptococcus* were more abundant in RA patients. Our results were similar to a previous study that reported that some *Lactobacillus* species might cause arthritis (Simelyte et al., 2003). *Eubacterium_hallii*, as one of the major butyrate producers (Louis et al., 2010), may play anti-rheumatic and anti-inflammatory effects by butyrate, which was proved that it inhibited arthritis and suppressed the expression of inflammatory cytokines in the CIA mice model (Hui et al., 2019). However, the change tendency of *Eubacterium_hallii* in our study was increased in patients with RA. Moreover, the abundance of *Bacteroides* was higher in healthy controls. This was consistent with a recent previous study that reported the decrement of a redox reaction-related gene of the genus *Bacteroides* in RA (Kishikawa et al., 2020).

Notably, in this study, we systematically analyzed the profile of fecal fungal biodiversity and community structure between RA and HCs. At the genus level, there were 401 specific fungal genera in the RA group, which were significantly more than the HC group. The LDA effect size analysis indicated that the seven discovered different fungal genera as a combination of biomarkers to diagnose RA included *Cryptococcus, Penicillium, Aspergillus, Cladosporium, Monascus, Candida*, and *Debaryomyces*. Especially, the *Candida* and *Debaryomyces* were more enriched in RA patients, which could be a potential biomarker for the prediction of early RA. Bishu et al. (2014). reported that there was clearly a trend toward increased susceptibility to *C. albicans* colonization in RA, and the risk of mucosal candidiasis in RA patients may increase by using biologic drugs selectively targeting the IL-23/IL-17 axis. Furthermore, Jain et al. (2021) discovered that yeast *Debaryomyces hansenii* were more likely to localize and be abundant within incompletely healed intestinal wounds of mice and inflamed tissue results from Crohn’s disease patients. Another study also confirmed that *Debaryomyces hansenii* could control the proliferation of opportunistic bacteria in the mucosa of intestinal microbiota disorder mice (Zeng et al., 2019). Although our results analyzed the fungal profile and changing trend in RA patients, the relationship of fungi with RA disease and the biological functions of fungi are still unclear and needs further exploration.

A recent study has reported that aberrant gut microbiota alters the host metabolome and impacts renal failure in humans, and gut microbiota is an important determinant of the host fecal and serum metabolic landscape (Wang et al., 2020b). A previous study has suggested that the serum metabolome can be impacted by dysbiosis of the human gut microbiota, *and Prevotella copri* and *Bacteroides vulgatus* are identified as the main species driving the association between biosynthesis of branched-chain AAs and insulin resistance (Pedersen et al., 2016). In our study, we analyzed the fecal bacterial community and fungal community structure and species diversity of RA patients and healthy controls and performed a functional predictive analysis of the identified differential communities. The results showed that plasma amino acid metabolism was associated with changes in gut microbes in RA patients. Especially, the arginine and proline metabolism pathways were significantly affected by microbiota changes. The expression of the metabolite L-Arginine was increased in RA patients and positively correlated with *Rhodotorula* in fungi. D-Proline levels were significantly positively correlated with abundances of *Agathobacte*r, *Roseburia*, and *Cladosporium*, and negatively correlated with *Candida*. *Escherichia-Shigella* abundance was positively correlated with plasma Creatine levels, while *Bacteroides* genus was negatively correlated with Creatine. Our results are consistent with those of a previous study that found that *Megamonas* was decreased in RA patients, which participates in the metabolism of carbohydrate fermentation into SCFAs (Feng et al., 2019). The functional predictive analysis results suggested that differential genera affect the host metabolic pathway of L-threonine biosynthesis, L-serine and glycine biosynthesis I, and “arginine, ornithine, and proline interconversion” through a variety of enzymes, such as choline dehydrogenase, D-amino-acid oxidase, glycine amidinotransferase, ornithine aminotransferase, lysophospholipase, choline-phosphate cytidylyltransferase, arginase, and phospholipase A (2), which from the functional prediction is based on a differential microbial genus. Interestingly, the functional prediction results showed a higher abundance of choline dehydrogenase, glycine amidinotransferase, phospholipase A (2), lower abundance of ornithine aminotransferase, and lysophospholipase, in bacteria of the RA group compared with HC group, however, the abundance of these enzymes was the exact opposite in fungi of RA patients.

There were several limitations. First, the critical role of metabolites biomarkers and microbial biomarkers need to be validated further. Second, though the glycine, serine, and threonine metabolism could help us draw a positive conclusion that they could be considered as a bridge between plasma and microbial metabolomes, the pathological mechanism from genotype to phenotype is not clear. Third, the untargeted metabolomics and microbiome were not using the same batch of personnel in the healthy control group, and a cautious interpretation of our result was needed when applying our model to other populations.

## Conclusion

In conclusion, we provided a list of metabolites, bacteria, and fungus, whose abundance changed in RA as potential biomarkers and built plasma metabolic/microbe-markers-based models for potentially clinical diagnosis of RA. The function prediction analysis of plasma metabolomics, intestinal bacteria, and intestinal fungi displayed that RA disease was related to changes in arginine and proline metabolism and glycine, serine, and threonine metabolism. Gut microbiome analysis combined with plasma metabolomics can shed new light on the pathogenesis of RA.

## Data availability statement

The data presented in the study are deposited in the NGDC database, accession number PRJCA011639.

## Ethics statement

The study was reviewed and approved by the Medical Ethics Committees of Dazhou Central Hospital.

## Author contributions

FZ, SL, and JZ contributed conception and design of the study. All authors were involved in organizing the database, performing the statistical analysis, drafting the article, and revising it critically for important intellectual content, agreed to be accountable for all aspects of the work and approved the final version to be published.
