# Supplementary material for: The change of plasma metabolic profile and gut microbiome dysbiosis in patients with rheumatoid arthritis
Source: Front Microbiol. 2022 Oct 18;13:931431. doi: 10.3389/fmicb.2022.931431 (PMC9623673; doi:10.3389/fmicb.2022.931431)
Supplement: Supplementary file 1 [file Data_Sheet_1.PDF]

Supplementary Material

1 Supplementary Methods

1.1 Non-targeted metabolomic analysis by UNPLC/Q-TOF-MS

Plasma sample extraction methods

After the sample is slowly thawed at 4°C, take an appropriate amount of sample and add it to the pre-cooled methanol/acetonitrile/water solution (2:2:1, v/v), Vortex to mix, sonicate at low temperature for 30 minutes, let stand at -20°C for 10 minutes, centrifuge at 14000 g at 4°C for 20 minutes, take the supernatant and vacuum dry. For mass spectrometry analysis, add 100 μL of acetonitrile aqueous solution (acetonitrile: water =1:1, v/v) to reconstitute, vortex, centrifuge at 14000 g at 4°C for 15 min.

Non-target LC-MS/MS Analysis

According to the instruction, we used an UHPLC (1290 Infinity LC, Agilent Technologies) coupled to a quadrupole time-of-flight (AB Sciex TripleTOF 6600) for HILIC separation, and all samples were analyzed by using a 2.1 mm × 100 mm ACQUIY UPLC BEH 1.7μm column (waters, Ireland). The A = 25 mM ammonium acetate and 25 mM ammonium hydroxide in water and B = acetonitrile were contained in the mobile phase in both ESI positive and negative modes. In ESI positive mode, the mobile phase contained A = water with 0.1% formic acid and B = acetonitrile with 0.1% formic acid; and in ESI negative mode, the mobile phase contained A = 0.5 mM ammonium fluoride in water and B = acetonitrile.

The gradient was 1%B for 1.5 min and was linearly increased to 99% in 11.5 min and kept for 3.5 min. Then the gradients were at a flow rate of 0.3 mL/min, and the column temperatures were kept constant at 25°C. The ESI source conditions were set as follows: Ion Source Gas1 (Gas1) as 60, Ion Source Gas2 (Gas2) as 60, curtain gas (CUR) as 30, source temperature: 600°C, IonSpray Voltage Floating (ISVF)± 5500 V. In MS only acquisition, the instrument was set to acquire over the m/z range 60-1000 Da, and the accumulation time for TOF MS scan was set at 0.20 s/spectra. The instrument was set to acquire over the m/z range 25-1000 Da, and the accumulation time for product ion scan was set at 0.05 s/spectra in auto MS/MS acquisition. The collision energy (CE) was fixed at 35 V with ± 15 eV and the declustering potential (DP) was 60 V (+) and −60 V (−); exclude isotopes within 4 Da, the candidate ions to monitor per cycle: 10.

1.2 Statistical analysis

Before importing into freely available XCMS software, we used ProteoWizard MSConvert to make the raw MS data (wiff.scan files) converted to MzXML files. For peak picking, these following parameters, including centWave m/z = 25 ppm, peakwidth = c (10, 60), prefilter = c (10, 100), were set up. For peak grouping, bw = 5, mzwid = 0.025, minfrac = 0.5 were used.

After normalized to total peak intensity, the processed data were analyzed by R package (ropls), where it was subjected to multivariate data analysis, including Pareto-scaled principal component

analysis (PCA) and orthogonal partial least-squares discriminant analysis (OPLS-DA). The 7-fold cross-validation and response permutation testing was used to evaluate the robustness of the model. The variable importance in the projection (VIP) value of each variable in the OPLS-DA model was calculated to indicate its contribution to the classification. Metabolites with the VIP value >1 and fold change higher 3/2 or lower 2/3 (p value < 0.05) was further applied to Student's t-test at univariate level to measure the significance of each metabolite.

To show the relationship and differences between metabolites, the hierarchical cluster were performed. The multidimensional statistical analysis, including PCA, PLS-DA and OPLS-DA, were also performed to help cluster the metabolites. Besides, permutation test was performed for the validation of the mode. Metabolic pathway (impact < 0.2, -log10(p) value > 1.5) and greater metabolic abundance, were identified by KEGG database.

1.3 Fecal sample DNA extraction and Illumina MiSeq sequencing

According to the manufacturer's protocol, total DNA was extracted from fecal samples. All DNA samples were quality checked, and the concentration was quantified by NanoDrop 2000 spectrophotometers (Thermo Fisher Scientific, Wilmington, DE, USA). Bacterial 16S rRNA gene fragments (V3-V4) were amplified from the extracted DNA using the primers 338F ACTCCTACGGGAGGCAGCAG and 806R GGACTACHVGGGTWTCTAAT, and fungal ITS gene fragments were amplified from the extracted DNA using the primers ITS1F CTTGGTCATTTAGAGGAAGTAA and ITS2R GCTGCGTTCTTCATCGATGC.

The following PCR conditions were used: 30 s at 95°C, 30 s at 55°C, and 45 s at 72°C for 37 cycles. Bacterial 16S rRNA PCR was performed with 4 μL 5×FastPfu Buffer, 2 μL 2.5 mM deoxynucleoside triphosphates (dNTPs), 0.8 μL of forward primer (5 μM), 0.8 μL *reverse primer (5 μM)*, 0.4 μL FastPfu Polymerase, 0.2 μL BSA, and 10 ng template DNA, and the volume was brought to 20 μL with ddH_2O. Fungal ITS PCR was performed with 2 μL 10× buffer, 2 μL 2.5 mM deoxynucleoside triphosphates (dNTPs), 0.8 μL of forward primer (5 μM), 0.8 μL *reverse primer (5 μM)*, 0.2 μL rTaq Polymerase, 0.2 μL BSA, and 10 ng template DNA, and the final volume as brought to 20 μL using ddH_2O. Agarose gel electrophoresis was performed to verify the size of amplicons. Amplicons were subjected to paired-end sequencing on an Illumina MiSeq sequencing platform using a PE250 kit.

1.4 Amplicon sequence processing and analysis

After demultiplexing, the resulting sequences were merged with FLASH (v1.2.11) and quality filtered with fastp (0.19.6). Then the high-quality sequences were de-noised using DADA2 plugin in the Qiime2 (version 2020.2) pipeline with recommended parameters, which obtains single nucleotide resolution based on error profiles within samples. DADA2denoised sequences are usually called amplicon sequence variants (ASVs). To minimize the effects of sequencing depth on alpha and beta diversity measure, the number of sequence from each sample was rarefied to 4000, which still yielded an average Good's coverage of 97.90%. Taxonomic assignment of ASVs was performed using the Naive bayes consensus taxonomy classifier implemented in Qiime2 and the SILVA 16S rRNA and ITS database.

2 Supplementary Figures and Tables

2.1 Supplementary Figures

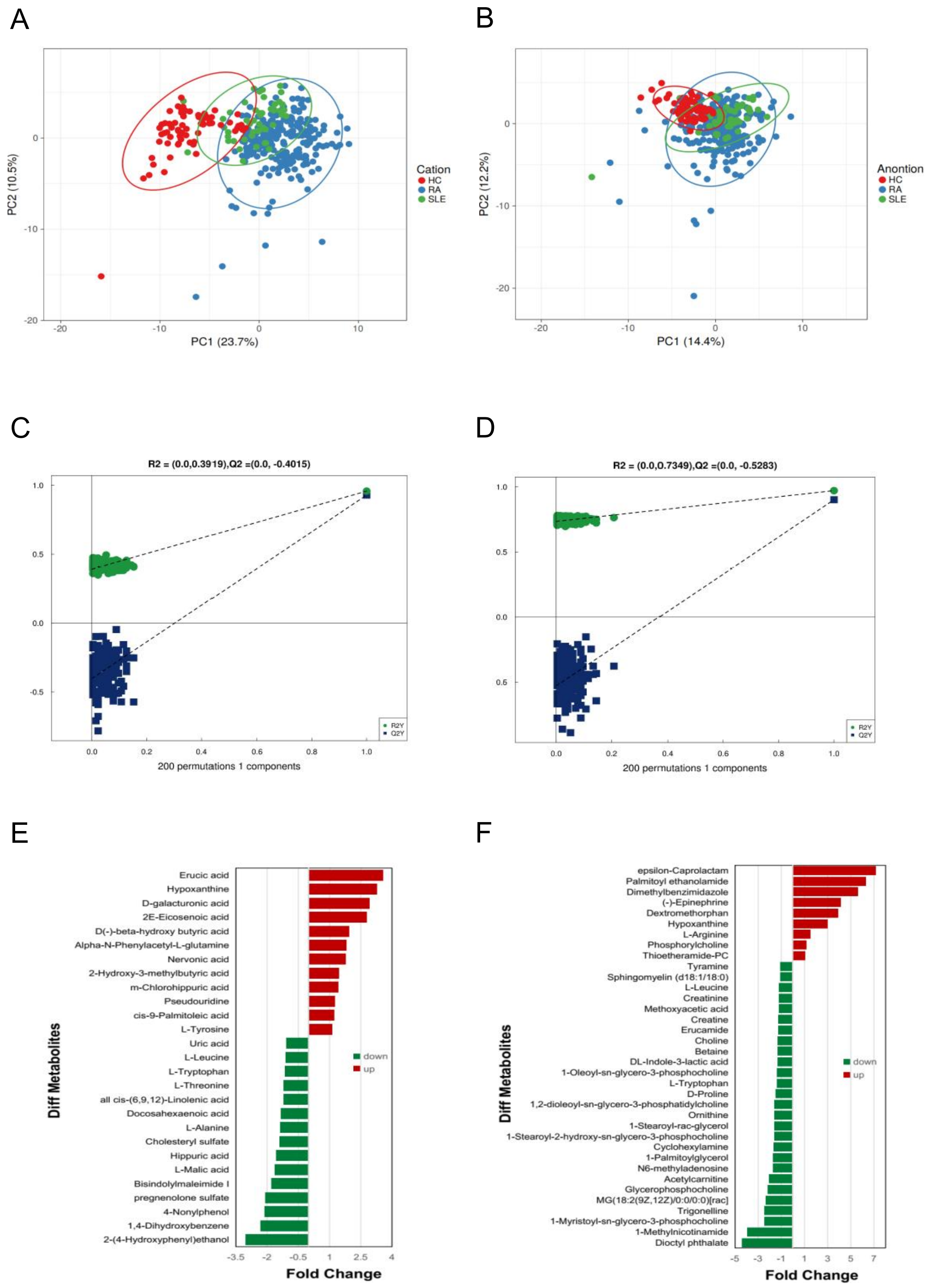

Supplementary Figure 1. The PCA and difference analysis of plasma metabolite at anion and cation. A, C. The Cation PCA diagram. B, D. Anion PCA diagram. E. The differential metabolites in Cation. F. The differential metabolites in Anion. Abbreviation: RA, Rheumatoid Arthritis; HC, healthy control; SLE, Systemic lupus erythematosus.

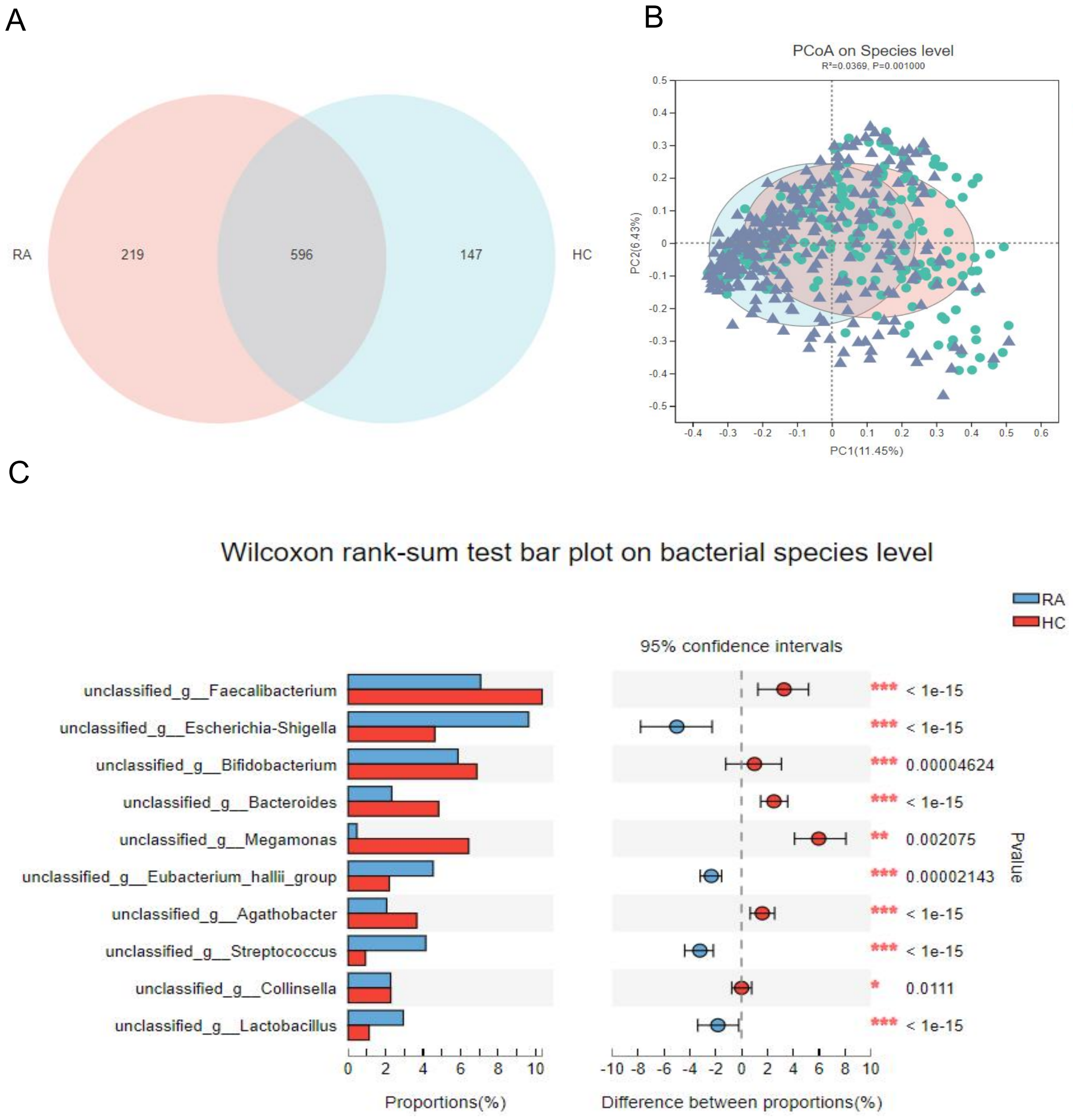

Supplementary Figure 2. The differential bacterial flora based on 16s rRNA (on species level) between RA and HC. A. The Venn plot. B.The PCoA analysis. C. The top 10 significant bacteria. Abbreviation: RA, Rheumatoid Arthritis; HC, healthy control.

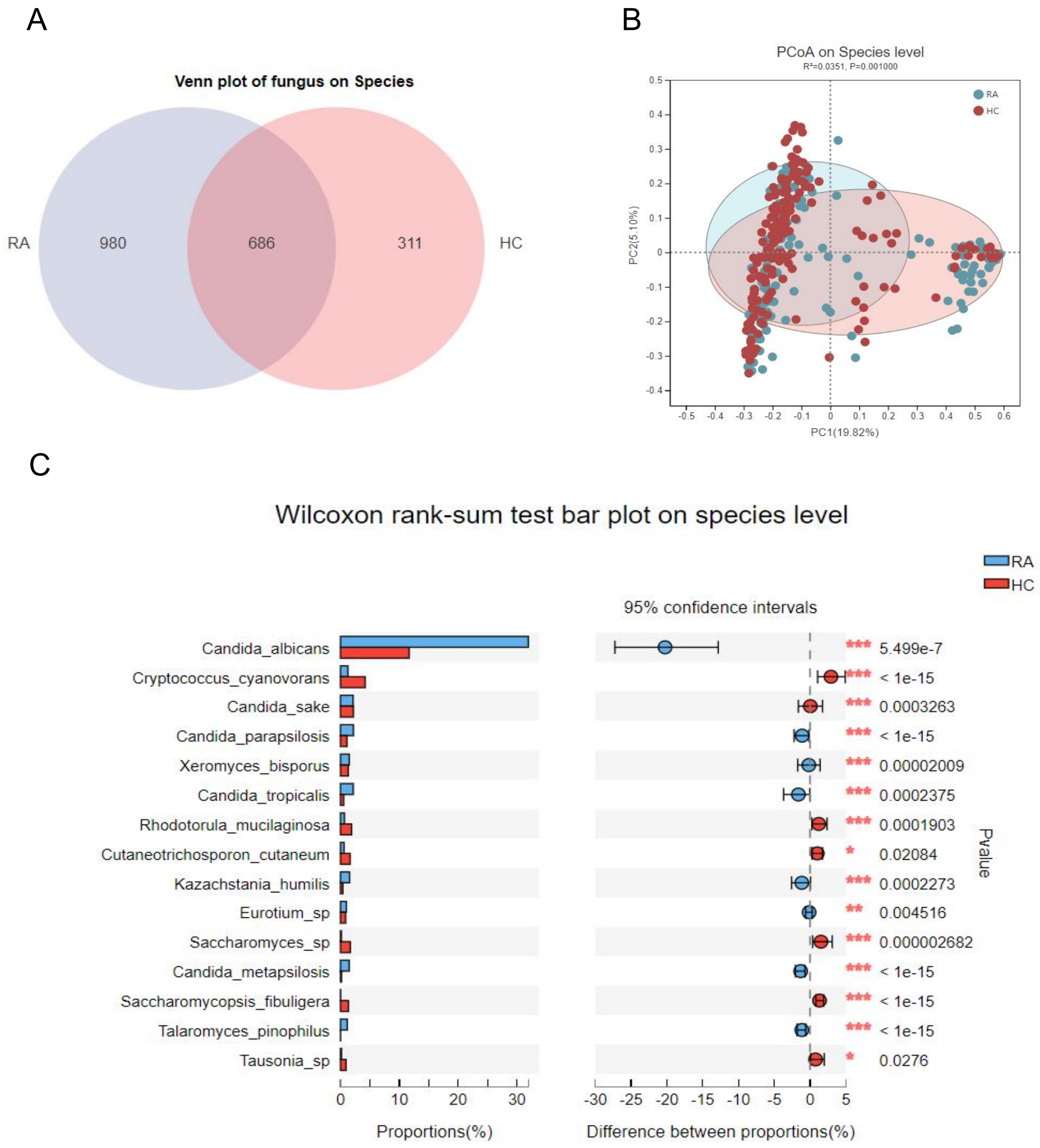

Supplementary Figure 3. The differential fungal flora based on ITS (on species level) between RA and HC. A. The Venn plot between groups. B.The PCoA analysis. C. The top 15 significant differential fungal. Abbreviation: RA, Rheumatoid Arthritis; HC, healthy control.

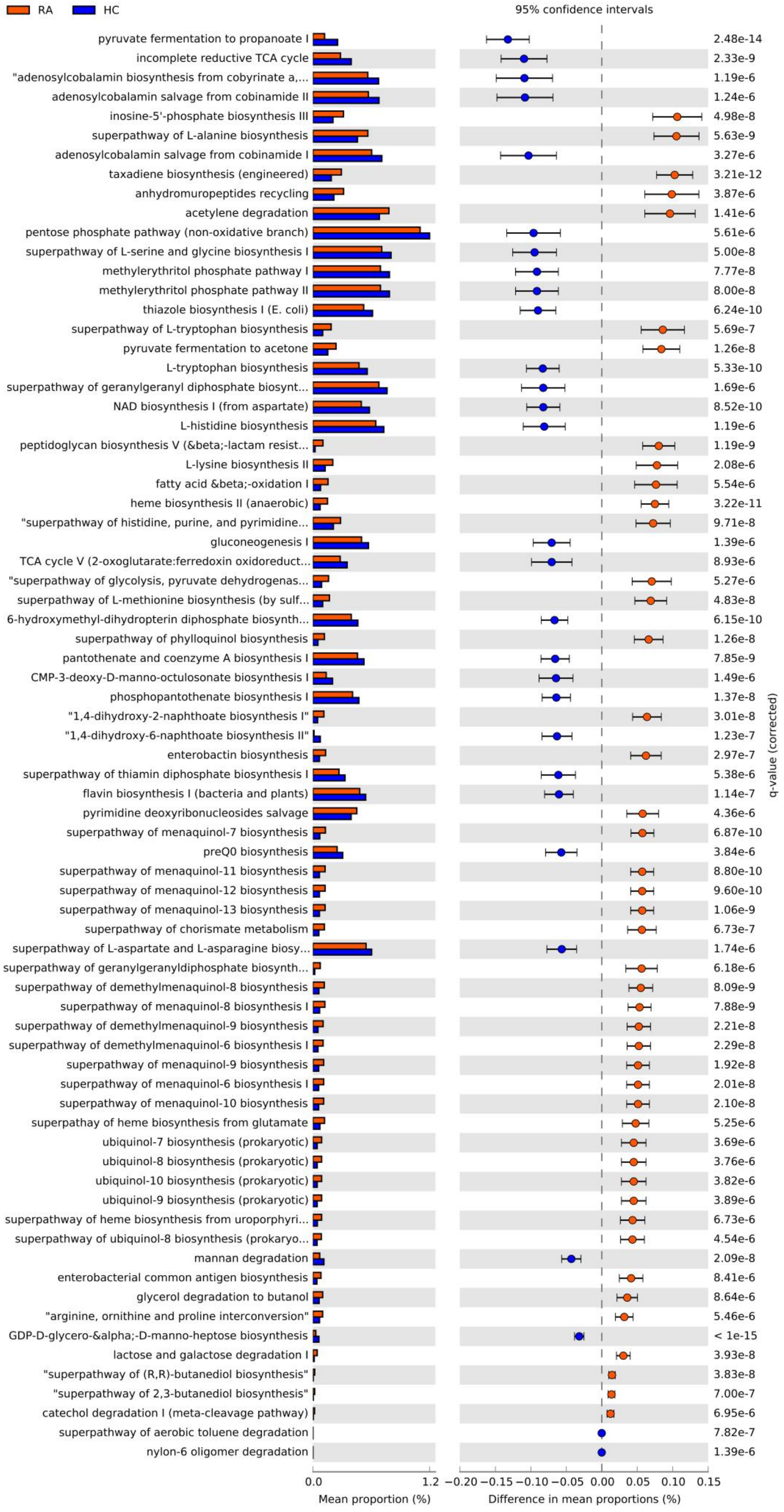

Supplementary Figure 4. The predicted pathways based on all differential bacterial flora (on genus level) between RA and HC.

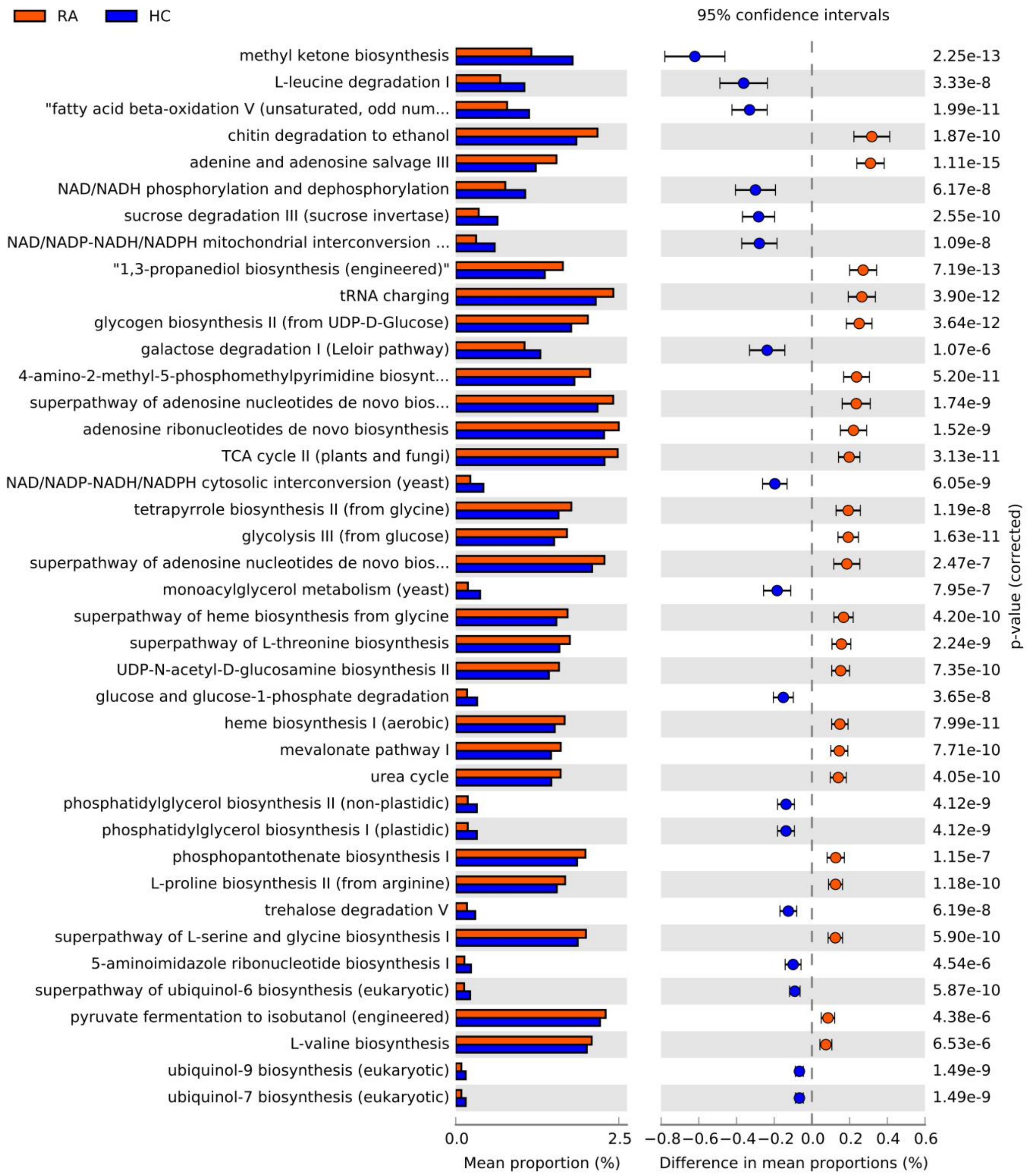

Supplementary Figure 5. The predicted pathways based on all differential fungal flora (on genus level) between RA and HC.

Supplementary Table 1. The 10 metabolites identified that can discriminate RA patients from healthy controls.

Metabolite/pathway	ESI mode	VIP	Fold change	p-value	m/z	rt(s)
Arginine and proline metabolism						
L-Arginine	Pos	6.8670	1.5239	7.29975E-10	175.1193	529.1905
Creatine	Pos	2.2664	0.7975	2.12054E-05	132.0767	342.5200
D-Proline	Pos	3.5828	0.6615	5.34941E-26	116.0707	306.7890
Ornithine	Pos	1.2194	0.6224	7.97946E-18	133.0964	511.3120
Glycine, serine and threonine metabol ism						
Choline	Pos	5.0871	0.7592	3.85925E-18	104.1073	272.9775
Betaine	Pos	1.2614	0.7530	4.15391E-07	118.0858	295.7850
L-Threonine	Neg	1.6641	0.8249	1.10191E-09	118.0504	350.2315
Glycerophospholipid metabolism						
LysoPC(18:0)	Pos	3.6474	0.6155	1.80358E-23	568.3364	188.972
Phosphorylcholine	Pos	1.6486	1.1798	0.002436806	184.0727	61.1365
Glycerophosphocholine	Pos	3.8177	0.4608	2.06775E-41	258.1098	382.083

Abbreviation: RA, Rheumatoid Arthritis; LysoPC(18:0),1-Stearoyl-2-hydroxy-sn-glycero-3-phosphocholine.
